# Echocardiographic analysis of dogs before and after surgical treatment of brachycephalic obstructive airway syndrome

**DOI:** 10.3389/fvets.2023.1148288

**Published:** 2023-05-04

**Authors:** Maja Brložnik, Alenka Nemec Svete, Vladimira Erjavec, Aleksandra Domanjko Petrič

**Affiliations:** Small Animal Clinic, Veterinary Faculty, University of Ljubljana, Ljubljana, Slovenia

**Keywords:** dog, brachycephalic, BOAS, echocardiography, obstructive sleep apnea

## Abstract

Brachycephalic dogs with brachycephalic obstructive airway syndrome (BOAS) are a valuable animal model for obstructive sleep apnea (OSA) in humans. Clinical signs of upper airway obstruction improve after surgical treatment of BOAS, but the impact of surgery on morphology and function of the heart has not been studied. Therefore, we aimed to compare the echocardiographic variables of dogs before and after surgical treatment of BOAS. We included 18 client-owned dogs with BOAS (7 French Bulldogs, 6 Boston Terriers, and 5 Pugs) scheduled for surgical correction. We performed a complete echocardiographic examination before and 6 to 12 (median 9) months after surgery. Seven non-brachycephalic dogs were included in the control group. After surgery, BOAS patients had a significantly (*p* < 0.05) larger left atrium to aortic ratio (LA/Ao), left atrium in the long axis index, and thickness of the left ventricular posterior wall in diastole index. They also had a higher late diastolic annular velocity of the interventricular septum (Am) and increased global right ventricular strain and left ventricular global strain in the apical 4-chamber view, as well as a higher caudal vena cava collapsibility index (CVCCI). Before surgery, BOAS patients had a significantly lower CVCCI, Am, peak systolic annular velocity of the interventricular septum (Si), and early diastolic annular velocity of the interventricular septum (Ei) compared to non-brachycephalic dogs. After surgery, BOAS patients had a smaller right ventricular internal diameter at base index, right ventricular area in systole index, mitral annular plane systolic excursion index, and tricuspid annular plane systolic excursion index, as well as lower values of Am, Si, Ei, and late diastolic annular velocity of the interventricular septum, and a larger LA/Ao compared to non-brachycephalic dogs. Significant differences between BOAS patients and non-brachycephalic dogs indicate higher right heart pressures and decreased systolic and diastolic ventricular function in BOAS dogs, which is in accordance with the results of studies in OSA patients. In parallel with the marked clinical improvement, right heart pressures decreased, and right ventricular systolic and diastolic function improved after surgery.

## Introduction

Brachycephaly, the shortening and/or flattening of the facial skeleton, is a mutation that occurs by selection in some dog breeds (1). Despite well-documented health problems related to conformation, brachycephalic dogs are becoming more and more popular (1, 2). Brachycephalic obstructive airway syndrome (BOAS) is a progressive disorder defined by primary anatomic abnormalities of the airways, such as narrowed nostrils, abnormal conchal growth, an elongated and thickened soft palate, deformity of the pharyngeal soft tissues, and a hypoplastic trachea. These abnormalities cause resistance to airflow and dyspnea, resulting in increased intraluminal pressure during inspiration and secondary soft tissue deformities manifested as pharyngeal hyperplasia, tonsillar hyperplasia, everted laryngeal saccules, and laryngeal collapse (1, 3–5). Extreme brachycephaly breeding selection has exacerbated upper airway stenosis, affecting younger dogs (1, 6).

Brachycephalic dogs are a valuable animal model for patients with obstructive sleep apnea (OSA), a sleep breathing disorder characterized by repeated transient cessations of breathing due to repeated intermittent episodes of upper airway obstruction, causing apneas or hypopneas, oxygen desaturations, and micro awakenings (7–12). In OSA, loss of pharyngeal dilator muscle tone at the onset of sleep leads to recurrent pharyngeal collapse and apnea, with greater risk in individuals with an abnormally narrowed or collapsible pharynx (8). Upper airway collapse and increased upper airway resistance cause changes in transpleural and intrathoracic pressure gradients, resulting in substantial increases in preload and afterload of the heart and atrial stretch, enlargement, remodeling, and fibrosis (9). Advanced age is one of the predisposing factors (9, 13); however, OSA is also a common condition in pediatrics; it is a hallmark in children with adeno-tonsillar hypertrophy (14–18).

Clinical signs of BOAS, which include stertor, stridor, panting, disturbed sleep patterns, exercise and heat intolerance, gagging, regurgitation, dyspnea, cyanosis, hyperthermia, and syncope, improve with corrective surgical treatment of BOAS (3, 19–23). However, no studies have been published on whether rhinoplasty and folded-flap palatoplasty affect cardiac morphology and function. Evaluation of right ventricular function 30 and 60 days after rhinoplasty showed that the elevated values of fractional area change of the right ventricle normalized 60 days after the procedure (24). Therefore, we aimed to compare echocardiographic variables in brachycephalic dogs before and six to 12 months after surgical correction of BOAS and also to compare them with echocardiographic variables in non-brachycephalic control dogs.

## Materials and methods

### Dogs

The brachycephalic group consisted of 18 client-owned dogs with BOAS who were scheduled for surgical correction of BOAS (rhinoplasty and folded-flap palatoplasty) and were offered an echocardiographic examination. Seven non-brachycephalic asymptomatic dogs with normal cardiac auscultation were included in the control group. The owners of the dogs included in this study have signed an informed agreement form before enrolment.

### Grading the severity of the disease

The severity of BOAS is graded according to obstruction at different anatomical levels, such as the nasopharynx, larynx, conchae and turbinates, pharyngeal folds, tonsils, and macroglossia (25, 26). The dogs were graded as 1, 2, or 3 on the basis of the reduction of the laryngeal airway radius after the soft palate was gently pulled rostrally to a position similar to the situation after folded-flap palatoplasty. All gradings and surgical treatments were conducted by the same experienced surgeon (V.E.).

### Rhinoplasty and folded-flap palatoplasty

Patients were fasted for 14 h prior to anesthesia. They received the following medications: 1 mg/kg maropitant i.v. (Prevomax 10 mg/ml, Eurovet Animal Health B.V., Bladel, Netherlands), 1 mg/kg pantoprazole i.v. (Nolpaza 40 mg, Krka, Novo Mesto, Slovenia), 0.2 mg/kg metoclopramide i.v. (Vomend 5 mg/ml, Eurovet Animal Health BV, Bladel, Netherlands) and 0.1 mg/kg dexamethasone i.m. (Dexamethason Krka 4 mg/ml, Krka, Novo Mesto, Slovenia). They were preoxygenated with 100% oxygen before premedication with 0.2 mg/kg butorphanol (Butomidor 10 mg/ml, Richter Pharma, Wels, Austria) mixed with 0.1 mg/kg midazolam i.v. (Midazolam Accord 1 mg/ml, Accord Healthcare, Poland Sp.z.o.o., Pabianice, Poland). For anesthesia induction and maintenance, propofol (Propomitor 10 mg/ml, Orion Corporation Orion Pharma, Espoo, Finland) was titrated (3–6 mg/kg) and oxygen administered by flow throughout the endoscopic examination of the nasopharynx, larynx, and trachea, which lasted approximately 10–20 min. After endoscopic examination and grading of the disease, patients were intubated in the sternal position, and anesthesia was maintained with isoflurane (Isoflurine 1,000 mg/g, Chemical Iberica PV, Spain). They received 4 mg/kg carprofen i.v. (Rycarfa 50 mg/ml, Krka, Novo Mesto, Slovenia) before the start of the surgical procedure. All dogs received fluids during the surgery (Hartmann’s solution 5 ml/kg/h, B Braun Melsungen AG, Melsungen, Germany). Surgery was performed on alar folds and soft palate. A vertical wedge excision aiming to involve the dorsomedial and caudal part of the ala was performed using a No. 11 scalpel blade. The wound edges were opposed with simple interrupted sutures using Glycomer 631 synthetic absorbable monofilament. In all patients, a folded-flap palatoplasty was performed as described elsewhere (19) with a Surgitron Radiolase II (Ellman International Inc., New York, United States). After recovering from anaesthesia, patients received 0.02 mg/kg buprenorphine i.v. (Bupredine Multidose 0.3 mg/ml, La Vet Beheer BV, TV Oudewater, Netherlands) and dexamethasone was repeated at the same dose 6 h after the first dose.

### Echocardiography

Two experienced echocardiographers (A.D.P, M.B.) performed a thorough echocardiographic examination of the right and left heart according to the guidelines (27–30) using a Vivid E9 (GE Healthcare, Europe) system and 1.75–3.5 or 4–10 MHz transducers. Simultaneously, a lead II ECG was recorded. We analyzed images with an offline Echopac workstation (GE Healthcare, Europe) in consensus by the two echocardiographers.

Echocardiographic measurements were taken from the right parasternal view, including linear dimensions of the left atrium and the ratio of the left atrium to aorta (LA/Ao), as well as M-mode measurements of the left ventricle. Right ventricular internal diameter measurements were obtained at the end of diastole. Linear and planar dimensions of the right atrium and right ventricle, spectral and color flow Doppler, and tissue Doppler of the ventricular walls at the annulus were measured from the left apical 4-chamber view. Mitral annular plane systolic excursion, tricuspid annular plane systolic excursion, and caudal vena cava at expiration and inspiration were measured in M-mode and were obtained from the left apical and cranial views. The global longitudinal strain of the left and right ventricle was obtained from the left apical view: aplax and 4-chamber and 2-chamber views using GE Echopac software. We averaged left ventricular global strain from three views: left apical long axis 5 chamber view, left apical long axis 4 chamber view (GSLV4ch), and left apical long axis 2 chamber view. We measured the right ventricular global strain (GSRV) in the left apical 4-chamber long axis view with the focus on the right ventricle.

We performed echocardiographic studies in conscious dogs during quiet breathing if possible. We averaged the echocardiographic variables from the measurements of three to five heartbeats.

Echocardiographic re-examination was performed 6 to 12 months after surgical treatment.

Weight-dependent variables were indexed: one dimensional as variable/weight^1/3^ and two-dimensional as variable/weight^2/3^ (31).

### Statistical analysis

For data analysis, we used commercial statistical software (IBM SPSS 25.0, Chicago, IL, United States). Data distributions were evaluated using the Shapiro–Wilk test. Based on these results, parametric or non-parametric tests were used to compare data between groups of dogs. Accordingly, to compare the measured parameters between BOAS patients and non-brachycephalic dogs before and after surgical treatment, we used the independent t-test in the case of normally distributed data or the Mann–Whitney test if the data were not normally distributed. In BOAS patients, we compared the measured parameters before and after surgical treatment with a paired t-test in the case of normally distributed data or with a Wilcoxon rank-sum test in the case of non normally distributed data.

We reported normally distributed data as means ± standard deviations and non-normally distributed data as medians and interquartile ranges (IQR; 25th to 75th percentiles). Statistical significance was set at *p* < 0.05.

## Results

### Age, weight, sex, and breed of the dogs

The age, weight, sex, and breed of the dogs diagnosed with BOAS are presented in Table 1. One dog was classified as BOAS grade 1, nine dogs as BOAS grade 2, and seven dogs as BOAS grade 3. The median follow-up time after surgical treatment was 9 months (range: 6 to 12 months).

**Table 1:** Characteristics of brachycephalic and non-brachycephalic dogs.

Variable (unit)	G1 Brachycephalic dogs before surgery (*n* = 18)	G2 Brachycephalic dogs after surgery (*n* = 18)	G3 Non-brachycephalic control dogs (*n* = 7)	*p* value G1/G2	*p* value G1/G3	*p* value G2/G3
Age (years)**	1.78 (0.88–3.65)	2.73 (1.74–4.47)	2.97 (2.67–4.49)	<0.001	0.053	0.397
Weight (kg)*	9.92 ± 2.41	10.69 ± 2.57	14.19 ± 3.61	0.014	0.002	0.012
Sex	12 males, 6 females		3 males, 4 females			
Breed	7 French bulldogs, 6 Boston terriers, 5 Pugs		5 Mixed breeds, 1 Tibetan terrier, 1 Border collie			

* Means ± standard deviation.

** Median (interquartile range: 25th to 75th percentile).

The median age of the brachycephalic dogs before surgery was not significantly different from that of the non-brachycephalic dogs (*p* = 0.053; Table 1).

There were three males and four females in the non-brachycephalic control group. The mean weight (±SD) of the brachycephalic dogs was significantly higher after surgery then before surgery (*p* = 0.014). The mean weight of the dogs in the non-brachycephalic group was significantly higher than that of the brachycephalic dogs before (*p* = 0.002) and after surgery (*p* = 0.012; Table 1).

### Echocardiographic variables In brachycephalic dogs before and after surgical treatment

After surgery, we identified a significantly larger LA/Ao ratio (*p* = 0.005), left atrium in long axis index (LALaxI; *p* = 0.018), and left ventricular posterior wall thickness in diastole index (*p* = 0.033) in BOAS patients (Table 2).

**Table 2:** 2D and M-mode echocardiographic variables in brachycephalic and non-brachycephalic dogs.

Variable	G1 Brachycephalic dogs before surgery (*n* = 18)	G2 Brachycephalic dogs after surgery (*n* = 18)	G3 Non-brachycephalic dogs (*n* = 7)	*p* value G1/G2	*p* value G1/G3	*p* value G2/G3
LA/Ao	1.50 ± 0.12	1.62 ± 0.13	1.44 ± 0.10	**0.005**	0.240	**0.002**
AoI (cm/kg^1/3^)	0.72 ± 0.08	0.69 ± 0.07	0.75 ± 0.09	0.190	0.463	0.120
LASaxI (cm/kg^1/3^)	1.08 ± 0.11	1.12 ± 0.08	1.08 ± 0.13	0.132	0.992	0.351
LALaxI (cm/kg^1/3^)	1.10 ± 0.15	1.19 ± 0.14	1.20 ± 0.08	**0.018**	0.110	0.879
IVSdI (cm/kg^1/3^)	0.34 ± 0.07	0.35 ± 0.07	0.39 ± 0.07	0.626	0.128	0.219
LVIDdI (cm/kg^1/3^)	1.38 ± 0.17	1.40 ± 0.18	1.40 ± 0.21	0.600	0.789	0.977
LVPWdI (cm/kg^1/3^)	0.33 ± 0.04	0.36 ± 0.05	0.36 ± 0.06	**0.033**	0.134	0.956
IVSsI (cm/kg^1/3^)	0.45 ± 0.09	0.48 ± 0.07	0.59 ± 0.23	0.282	0.173	0.258
LVIDsI (cm/kg^1/3^)	0.88 ± 0.12	0.91 ± 0.13	0.97 ± 0.17	0.368	0.166	0.369
LVPWsI (cm/kg^1/3^)	0.47 ± 0.06	0.47 ± 0.08	0.49 ± 0.05	0.957	0.350	0.496
FS (%)	36.2 ± 8.2	34.7 ± 6.0	31.3 ± 6.0	0.320	0.161	0.216
RVIDbasI (cm/kg^1/3^)	0.69 ± 0.13	0.54 ± 0.09	0.67 ± 0.16	**<0.001**	0.679	**0.015**
RVIDmidI (cm/kg^1/3^)	0.73 ± 0.17	0.69 ± 0.13	0.74 ± 0.16	0.234	0.910	0.428
RVIDlongI (cm^2^/kg^2/3^)	1.28 (1.17–1.42)	1.23 (1.08–1.35)	1.3 (1.16–1.39)	0.093	0.904	0.226
RVIDlong/RVIDmid	1.75 (1.58–1.94)	1.79 (1.58–1.86)	1.94 (1.58–2.15)	0.862	0.505	0.430
RVAdI (cm^2^/kg^2/3^)	0.77 ± 0.29	0.71 ± 0.21	0.89 ± 0.21	0.121	0.337	0.071
RVAsI (cm^2^/kg^2/3^)	0.39 (0.28–0.48)	0.32 (0.25–0.41)	0.56 (0.36–0.59)	0.065	0.084	**0.015**
FAC (%)	49.8 ± 9.9	52.5 ± 8.9	45.14 ± 5.43	0.443	0.250	0.054
RAAI (cm^2^/kg^2/3^)	0.74 ± 0.2	0.67 ± 0.16	0.75 ± 0.25	0.338	0.915	0.310
TAPSEI (cm/kg^1/3^)	0.53 (0.47–0.64)	0.49 (0.4–0.55)	0.61 ± 0.12	0.114	0.105	**0.022**
MAPSEI (cm/kg^1/3^)	0.39 ± 0.11	0.39 ± 0.07	0.47 ± 0.06	0.871	0.083	**0.031**
CVCII (cm/kg^1/3^)	0.25 ± 0.09	0.21 ± 0.05	0.22 ± 0.03	0.138	0.327	0.859
CVCEI (cm/kg^1/3^)	0.42 (0.37–0.47)	0.41 (0.35–0.45)	0.41 (0.36–1.51)	0.468	0.727	0.832
CVCCI (%)	41.57 ± 11.30	49.18 ± 7.90	49.43 ± 4.29	**0.049**	**0.022**	0.939

Echocardiographic variables are presented as means ± standard deviations or medians (interquartile range: 25th to 75th percentile). Right ventricular internal diameter measurements were obtained at the end of diastole. AoI = aortic diameter index, CVCEI = caudal vena cava at expiration index, CVCII = caudal vena cava at inspiration index, CVCCI = caudal vena cava collapsibility index = (CVCE–CVCI)/CVCE*100%, FAC = fractional area change of right ventricle, FS = fractional shortening of left ventricle, G1 = group 1 (brachycephalic dogs before surgery), G2 = group 2 (brachycephalic dogs after surgery), G3 = group 3 (non-brachycephalic control dogs), I = weight dependable variable index = one dimensional as variable/weight^1/3^ and two dimensional as variable/weight^2/3^, IVSdI = thickness of interventricular septum in diastole index, IVSsI = thickness of interventricular septum in systole index, LALaxI = diameter left atrium in long axis index, LASaxI = diameter of left atrium in short axis index, LA/Ao = ratio of left atrium to aorta in short axis, LVIDdI = left ventricular internal diameter in diastole index, LVIDsI = left ventricular internal diameter in systole index, LVPWdI = thickness of left ventricular posterior wall in diastole index, LVPWsI = thickness of left ventricular posterior wall in systole index, MAPSEI = mitral annular plane systolic excursion index, RAAI = right atrial area index, RVAdI = right ventricular area in diastole index, RVAsI = right ventricular area in systole index, RVIDbasI = right ventricular internal diameter at base index, RVIDmidI = right ventricular internal diameter in mid cavity index, RVIDlongI = right ventricular longitudinal diameter index, RVIDlong/RVIDmid = right ventricular longitudinal diameter to right ventricular internal diameter in mid cavity ratio, TAPSEI = tricuspid annular plane systolic excursion index. *p* values 0.05 - statistically significant.

**Figure 1:**
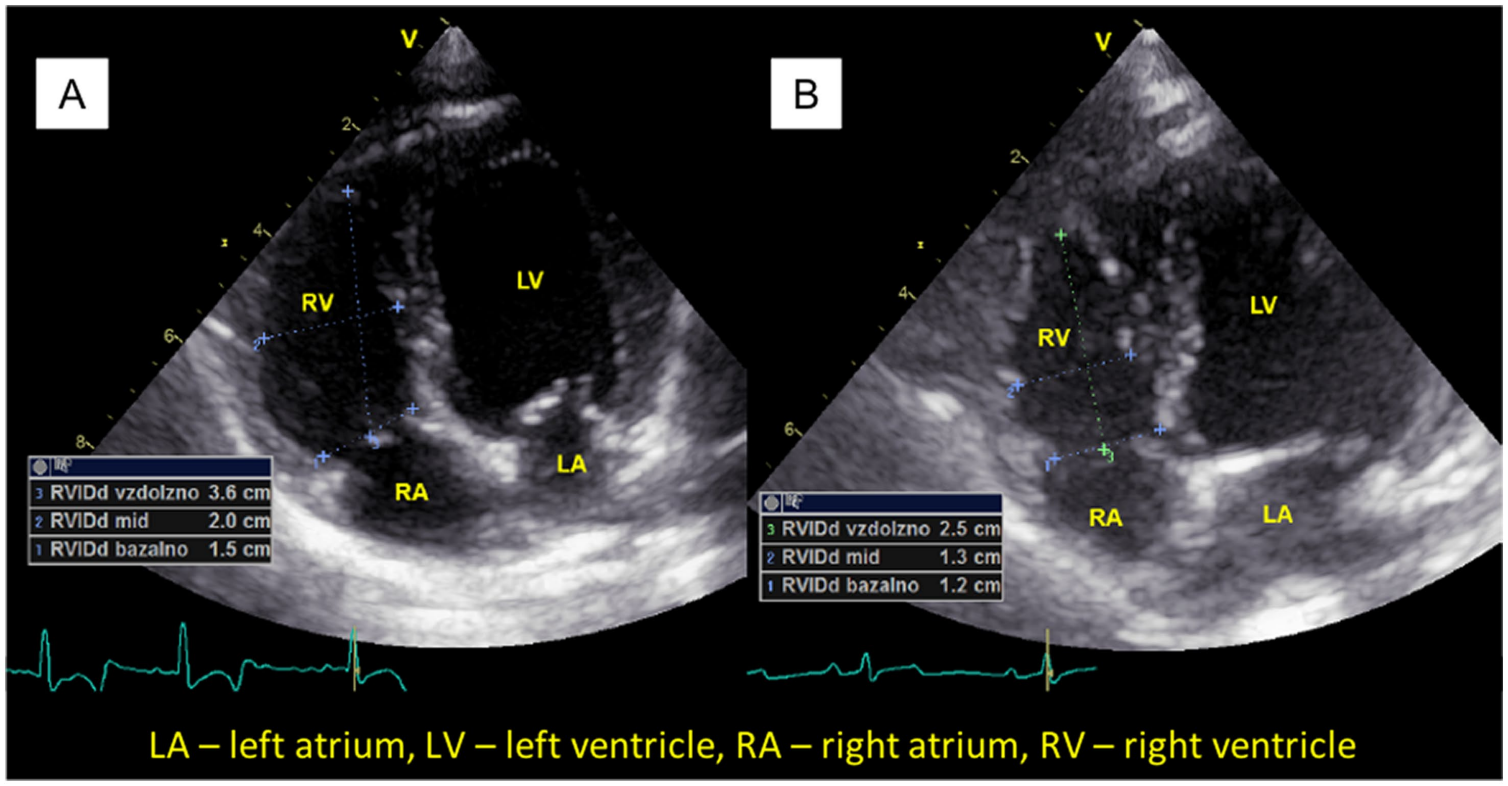
The right ventricular internal diameter at base index (RVIDbasI) was significantly larger in brachycephalic dogs before surgery **(A)** than after surgery **(B)**. Right ventricular internal diameter measurements were measured at the end of diastole. LA, left atrium; LV, left ventricle; RA, right atrium; RV, right ventricle.

After surgical treatment, the caudal vena cava collapsibility index (CVCCI) was significantly higher (*p* = 0.049), and the right ventricular internal diameter at base index (RVIDbasI; *p* < 0.001; Figure 1) was significantly smaller (Table 2).

**Figure 2:**
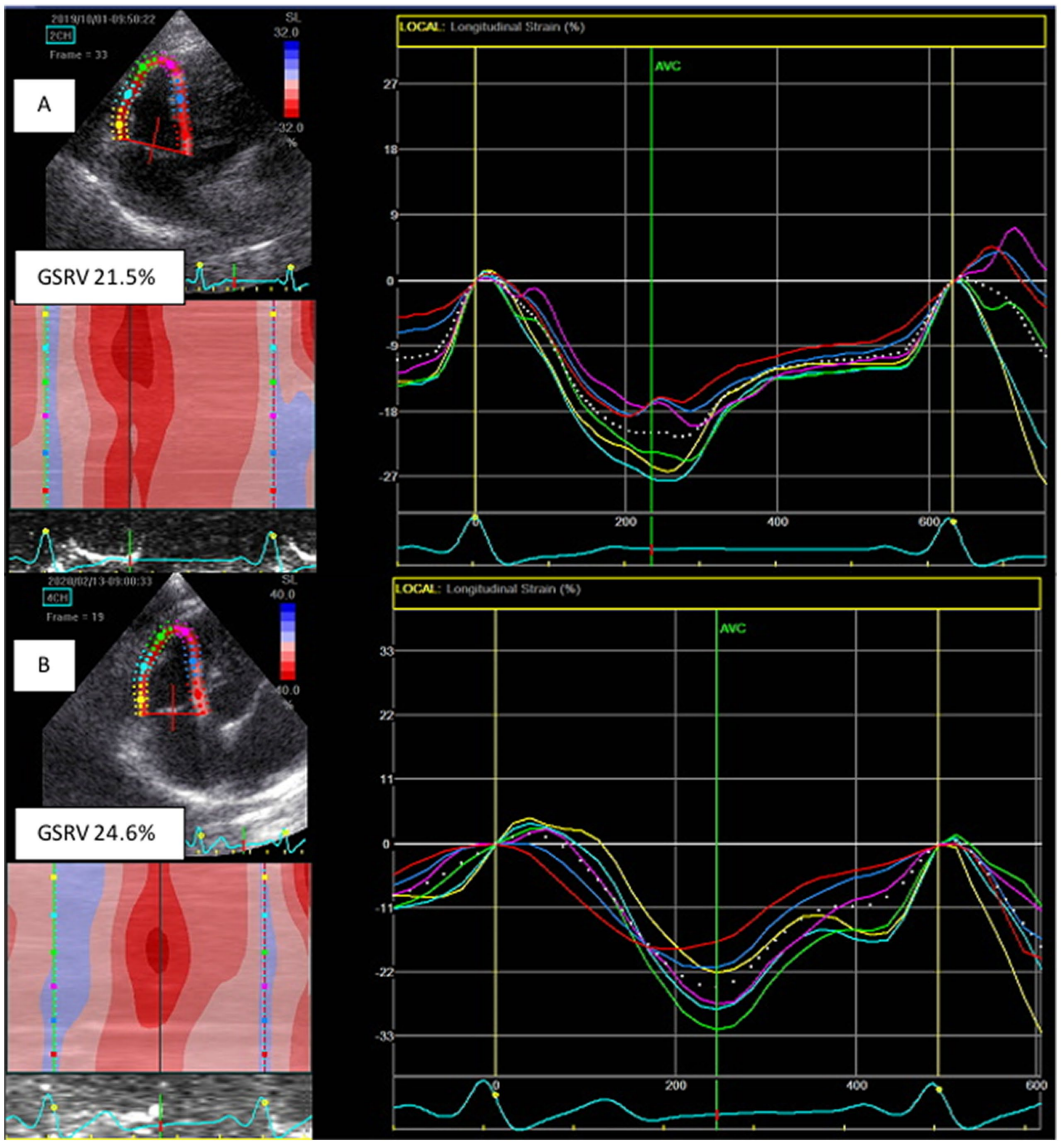
Global right ventricular strain (GSRV) was significantly lower in brachycephalic dogs before surgery **(A)** than after surgery **(B)**.

In addition, BOAS dogs had a significantly lower late diastolic interventricular annular velocity (Ai; *p* = 0.021) and higher GSRV (*p* = 0.033; Figure 2) and GSLV4ch after surgery (*p* = 0.020; Table 3).

**Table 3:** Doppler derived echocardiographic variables in brachycephalic and non-brachycephalic dogs.

Variable	G1 Brachycephalic dogs before surgery (*n* = 18)	G2 Brachycephalic dogs after surgery (*n* = 18)	G3 Non-brachycephalic dogs (*n* = 7)	*p* value G1/G2	*p* value G1/G3	*p* value G2/G3
MVE (m/s)	0.74 ± 0.15	0.74 ± 0.14	0.73 ± 0.10	0.907	0.893	0.850
MVA (m/s)	0.54 ± 0.08	0.51 ± 0.08	0.54 ± 0.09	0.304	0.972	0.471
MVE/A	1.40 ± 0.31	1.48 ± 0.26	1.37 ± 0.17	0.241	0.822	0.333
TVE (m/s)	0.68 ± 0.13	0.73 ± 0.11	0.68 ± 0.13	0.313	0.999	0.409
TVA (m/s)	0.44 ± 0.12	0.50 ± 0.12	0.49 ± 0.10	0.056	0.369	0.859
TVE/A	1.62 ± 0.22	1.51 ± 0.22	1.45 ± 0.35	0.051	0.179	0.612
MVE/TVE	1.12 ± 0.29	1.06 ± 0.23	1.10 ± 0.26	0.445	0.888	0.714
MVA/TVA	1.28 ± 0.40	1.10 ± 0.31	1.11 ± 0.17	0.052	0.302	0.895
Sm (cm/s)	8.29 ± 2.78	9.23 ± 2.81	10.16 ± 3.00	0.261	0.153	0.475
Em (cm/s)	8.45 (6.75–10.30)	9.40 (7.03–11.70)	10.5 (9.2–13.2)	0.542	0.084	0.364
MVE/Em (cm/s)	7.55 (6.75–10.10)	7.60 (6.50–9.30)	6.70 (6.0–7.30)	0.616	0.090	0.215
Am (cm/s)	6.52 ± 2.25	7.19 ± 1.76	9.83 ± 1.66	0.147	**0.002**	**0.002**
Si (cm/s)	6.91 ± 2.53	6.74 ± 2.78	11.47 ± 3.09	0.824	**0.001**	**0.001**
Ei (cm/s)	6.31 ± 1.87	6.29 ± 2.03	8.04 ± 1.24	0.975	**0.034**	**0.046**
MVE/Ei (cm/s)	10.5 (10.03–14.98)	12.3 (10.93–14.15)	9.7 (7.7–11.3)	0.948	0.074	**0.011**
Ai (cm/s)	6.45 ± 2.18	4.95 ± 1.37	8.11 ± 2.23	**0.021**	0.105	**<0.001**
St (cm/s)	10.24 ± 2.96	10.38 ± 3.57	14.42 ± 6.67	0.845	0.153	0.169
Et (cm/s)	9.98 ± 2.49	10.94 ± 2.03	10.07 ± 3.50	0.131	0.941	0.444
TVE/Et (cm/s)	7.11 ± 1.87	6.72 ± 1.33	7.23 ± 1.64	0.512	0.886	0.438
At (cm/s)	8.04 ± 1.83	8.16 ± 2.59	11.96 ± 4.49	0.826	0.062	0.070
GSAplax (%)	19.2 (15.9–22.8)	19.4 (15.8–21.1)	19.35 (17.78–21.68)	0.698	0.685	0.685
GSLV4ch (%)	18.30 ± 3.13	19.96 ± 4.33	18.13 ± 0.79	**0.020**	0.849	0.123
GSLV2ch (%)	19.77 ± 5.68	21.69 ± 5.24	20.20 ± 4.19	0.254	0.868	0.544
GSLV (%)	19.14 ± 3.56	20.07 ± 3.84	19.47 ± 1.63	0.226	0.829	0.720
GSRV (%)	19.36 ± 5.26	23.12 ± 5.59	24.02 ± 4.48	**0.033**	0.065	0.729

Echocardiographic variables are presented as means ± standard deviations or medians (interquartile range: 25th to 75th percentile). Ai = late diastolic annular velocity of the interventricular septum, Am = late diastolic annular velocity of the left ventricular free wall, At = late diastolic annular velocity of the right ventricular free wall, Ei = early diastolic annular velocity of the interventricular septum, Em = early diastolic annular velocity of the left ventricular free wall, Et = early diastolic annular velocity of the right ventricular free wall, G1 = group 1 (brachycephalic dogs before surgery), G2 = group 2 (brachycephalic dogs after surgery), G3 = group 3 (non-brachycephalic control dogs), GSAplax = left ventricular global strain in apical long axis 5 chamber view, GSLV4ch = left ventricular global strain in apical long axis 4 chamber view, GSLV2ch = left ventricular global strain in apical long axis 2 chamber view, GSRV = right ventricular global strain in apical long axis view, MVA = late diastolic mitral wave velocity, MVE = early diastolic mitral wave velocity, MVE/A = early to late diastolic mitral wave velocity ratio, MVA/TVA = late diastolic mitral wave velocity to early tricuspid wave velocity ratio, MVE/Ei = early diastolic mitral wave velocity to early diastolic annular velocity of the interventricular septum ratio, MVE/Em = early diastolic mitral wave velocity to early diastolic annular velocity of the left ventricular free wall ratio, Si = peak systolic annular velocity of the interventricular septum, Sm = peak systolic annular velocity of the left ventricular free wall, St = peak systolic annular velocity of the right ventricular free wall, TVA = late diastolic tricuspid wave velocity, TVE = early to late diastolic tricuspid wave velocity, TVE/A = early to late diastolic tricuspid wave velocity ratio. *p* values 0.05 - statistically significant.

Tricuspid regurgitation was documented in four dogs, all of which were Boston Terriers. The tricuspid regurgitation pressure gradient was considered as mild pulmonary hypertension (31–50 mm Hg) in three dogs before surgery, and although it decreased after surgery, we could not statistically assess the difference due to low number of dogs.

### Comparison of echocardiographic variables between brachycephalic dogs before surgical treatment and non-brachycephalic control dogs

In brachycephalic dogs before surgery, CVCCI was lower (*p* = 0.022) in comparison to dogs in the control group (Table 2). In brachycephalic dogs before surgery, the late diastolic annular velocity of the left ventricular free wall (Am; *p* = 0.002), peak systolic annular velocity of the interventricular septum (Si; *p* = 0.001), and early diastolic annular velocity of the interventricular septum (Ei; *p* = 0.034) were lower in comparison to non-brachycephalic control group dogs (Table 3).

### Comparison of echocardiographic variables between brachycephalic dogs after surgical treatment and non-brachycephalic control dogs

A larger LA/Ao (*p* = 0.002) and a smaller RVIDbasI (*p* = 0.015), right ventricular area in systole index (RVAsI; *p* = 0.015), mitral annular plane systolic excursion index (*p* = 0.031) and tricuspid annular plane systolic excursion index (*p* = 0.022) were observed in brachycephalic dogs after surgery compared to the dogs in the control group (Table 2). In addition, brachycephalic dogs had a lower Am (*p* = 0.002), Si (*p* = 0.001), Ei (*p* = 0.046) and Ai (*p* < 0.001), and a higher early diastolic mitral wave velocity (MVE) to Ei ratio (*p* = 0.011) than the dogs in the control group (Table 3).

## Discussion

This study is the first to investigate the effect of rhinoplasty and folded-flap palatoplasty on echocardiographic variables in brachycephalic dogs with BOAS. The brachycephalic dogs in our study belonged to three breeds that were already identified as the most commonly affected by BOAS: French Bulldogs, Pugs, and Boston Terriers (4, 32, 33).

Several echocardiographic variables differed significantly in dogs before and after BOAS surgical treatment: LA/Ao, LALaxI, CVCCI, RVIDbasI, RVAsI, Ai, GSRV, and GSLV4ch. In dogs after surgery, LA/Ao and LALaxI were larger than before surgery, which could be a consequence of lower right heart pressure after surgery. Right heart volume decreased after surgery (lower RVIDbas), and systolic ventricular function improved (smaller RVAsI and higher GSRV and GSLV4ch). Brachycephalic dogs had the same LA/Ao in comparison to the control group; however, after surgery, LA/Ao was significantly larger, most likely due to the above-mentioned decrease in right heart volume and pressure. The dogs gained weight after surgery, probably due to less respiratory distress or fewer gastrointestinal problems associated with BOAS. In human studies, with the obesity-induced hypoventilation syndrome and OSA were twice as likely to have a larger left atrial diameter than patients with OSA alone (34). In OSA patients, there is no agreement regarding left atrial dilatation (35). In several studies, left atrial structural and functional remodeling correlated to the severity of OSA (13, 34, 36–40), while in other studies, no correlation was found between left atrial dilation and the severity of OSA (41, 42). Compared to non-brachycephalic dogs, a significantly larger LA/Ao was demonstrated in different brachycephalic breeds (43–48). In our study, LA/Ao did not differ between brachycephalic dogs before surgery and non-brachycephalic dogs, which is inconsistent with previous studies (43–48). We suspect that the reason for this might be small number of dogs included, which were also only symptomatic dogs. In a recent publication, we found a significantly smaller left atrial size in French bulldogs with BOAS compared with French bulldogs without BOAS or non-brachycephalic dogs, the latter also being true for Pugs in that study (48).

One of the major risk factors for OSA in humans is obesity (34) and obesity worsens breathing in brachycephalic dogs with BOAS, as well (33). The effects of obesity on breathing include a lower respiratory minute volume with a concomitant respiratory rate elevation, exercise intolerance, and an arterial oxygen saturation decrease (33). Left ventricular hypertrophy even in the absence of hypertension, obesity, and diabetes was reported in OSA patients (12, 40, 49).

In brachycephalic dogs, CVCCI was higher after surgery, indicating lower right heart diastolic pressures. Furthermore, CVCCI after surgery did not differ from that of the non-brachycephalic dogs, indicating normalization of diastolic pressures, whereas in dogs before surgery CVCCI was higher than in non-brachycephalic dogs, suggesting higher right heart diastolic pressures in dogs before surgery. The reason for the marked fluctuations of intrathoracic pressure observed in our study are obstructive airways that increase venous return and lead to volume overload of the right ventricle (43). In advanced stages of OSA, pulmonary hypertension causes right ventricular pressure overload due to arterial hypoxemia and hypercapnia during sleeping (7). Hypoxic conditions lead to constriction of the pulmonary artery, and an elevated pulmonary vascular resistance influences the pressure overload of right ventricle, leading to hypertrophy and subsequent dilation (7). In our study, the tricuspid regurgitation pressure gradient was consistent with mild pulmonary hypertension in three dogs before surgery (17%), and although the pulmonary arterial pressure decreased in all three dogs after surgery, we were unable to statistically assess the difference due to small number of dogs involved. Morpho-functional changes due to anatomic abnormalities of the upper airway increase airflow resistance that may trigger an increase in pulmonary pressure due to hypoxic vasoconstriction (7). The presumed aftermath of vasoconstriction would be the right heart remodeling with the potential development of right heart failure (43, 50). In patients with OSA, pulmonary hypertension occurs in 12–70% depending on the severity of OSA (12).

In our study, RVIDbasI and RVAsI were smaller in brachycephalic dogs after surgery, which may indicate improved right ventricular systolic function after lowering intrathoracic pressure and less upper airway obstruction. However, RVIDbasI did not differ between brachycephalic dogs before surgery and non-brachycephalic dogs. We assume that right heart remodelling due to BOAS is happening gradually over the years, whereas dogs in our study were relatively young. In patients with moderate to severe OSA, right ventricular end-diastolic and end-systolic volume indices are higher compared to control subjects (12, 38). We believe that the reason for the difference between our study and the results obtained in OSA patients is probably the young age of the dogs in our study whereas severe OSA patients are usually older. Similarly, in a study including 22 French Bulldogs and six healthy Beagle dogs, right ventricular changes were statistically insignificant (43). In a study including 42 Pugs, no significant differences were found between dogs with BOAS and those without BOAS, except for peak pulmonary velocity; however, neither right heart dimensions nor tissue Doppler measurements were performed in this study (44). Failure to observe early morpho-functional changes in the right ventricle in our and other studies may imply the need for the use of magnetic resonance imaging as a more sensitive technique (51). Transthoracic echocardiography is the most valuable tool for evaluating canine cardiac structure and function, but it can be more difficult in brachycephalic dogs due to dorsoventral compression of the thorax, obesity, and narrow intercostal spaces (52). The right ventricle varies in size and motion with respiration, and right ventricular pressure is lower than left ventricular pressure; therefore, the right ventricle is more sensitive to increased intrathoracic pressure due to upper airway obstruction.

In our study, brachycephalic dogs had lower Am, Si, and Ei before surgery and lower Si, Am, and Ai after surgery than non-brachycephalic control dogs. Lower Si may suggest decreased systolic function of the heart, whereas lower Ei, Am, and Ai may indicate decreased diastolic function of the ventricles. In human patients, echocardiographic parameters such as right ventricular fractional area of change, tricuspid annular plane systolic excursion, and peak systolic annular velocity, as well as three-dimensional evaluation and global longitudinal strain, have been found to improve the assessment of right ventricular function, providing both diagnostic and prognostic data on various clinical conditions, including pulmonary hypertension and heart failure (53). In patients with OSA, right ventricular strain, right ventricular systolic strain rate, and right ventricular early diastolic strain all decrease with increasing OSA severity, whereas the right ventricular late diastolic strain rate increases (54). Tissue Doppler imaging is sensitive for identification of ventricular dysfunction; however, angle dependence and the undesirable effect of tethering forces limit its use (12). On the other hand, speckle tracking echocardiography can discern between active and passive wall motion and is therefore a better tool for the assessment of ventricular function (12).

In our study, we observed that brachycephalic dogs had higher GSRV and GSLV4ch after surgery. This suggest that ventricular function improves in these dogs following surgical intervention. Interestingly, there was no difference in GSRV and GSLV4ch between non-brachycephalic dogs and brachycephalic dogs before or after surgery. We hypothesize that the differences in global strain values between non-brachycephalic and brachycephalic dogs are small because of young age of brachycephalic dogs. In a study with 42 conscious healthy dogs, global longitudinal strain and strain rate showed a correlation with breed and weight (55). In humans, the estimation of right ventricular mechanics remains challenging due to the complex right ventricular shape and retrosternal location in the mediastinum, which complicates echocardiographic assessment of the right ventricular (56, 57). Similarly, in veterinary echocardiography, this is further complicated by various thoracic shapes present in different breeds. However, in humans, longitudinal strain of the right ventricle is a useful diagnostic tool for detecting subclinical myocardial dysfunction (55). Additionally, one study has shown that right ventricular longitudinal strain has high predictive value in several cardiovascular diseases, including heart failure, pulmonary hypertension, mitral valve disease, OSA, and congenital cardiac disease (35). In patients with OSA, right ventricular preload is increased by negative intrathoracic pressure, while pulmonary vasoconstriction is caused by apnea-induced hypoxia and this increases right ventricular afterload (8). These forces expand the right ventricular, and cause the interventricular septum to shift during diastole, impeding filling of the left ventricular and decreasing stroke volume (8). Additionally, cardiac contractility and diastolic relaxation may be directly affected by hypoxia during OSA (8).

Previous reports of OSA in a canine model have displayed that each episode of hypoxia decreases left ventricular diastolic function by elevating left ventricular afterload, and that left ventricular systolic dysfunction occurs as early as 3 months (50, 58). In humans, OSA-induced systolic and diastolic dysfunction appears to show a similar pattern; however, it develops over a longer time period, initially affecting diastolic function and leading to systolic dysfunction only after prolonged exposure, likely over 10 years (12, 51, 59). Literature reports on left ventricular ejection fraction and fractional shortening in OSA patients are inconsistent. Some publications show normal left ventricular ejection fraction in patients with OSA (40, 60), and some studies report no significant differences in left ventricular ejection fraction or fractional shortening between different severities of OSA (40, 61). However, two studies found that the severity of OSA was associated with a decrease in left ventricular ejection fraction (62, 63). Significantly decreased values of global left ventricular longitudinal strain and basal, mid, and apical strain values were reported in patients with severe OSA compared to the other groups (62). In another study, only tissue Doppler-derived variables (and not mitral flow pattern) were significantly different in OSA patients; early diastolic annular velocity of the left ventricular free wall (Em) was significantly lower in patients with OSA than in control subjects, which indicates decreased diastolic function (61, 64). Peak systolic annular velocity, an indicator of ventricular systolic function, did not differ significantly between OSA patients and control subjects, which is consistent with the controversial reports on left ventricular ejection fraction in OSA mentioned above (12, 40, 62). The mean MVE/Em ratio, an estimate of left ventricular end-diastolic pressures, is higher in individuals with moderate to severe OSA than in patients with mild disease and/or healthy individuals (65–68). In our study, MVE/Em did not differ between brachycephalic dogs before and after surgery, nor between brachycephalic and non-brachycephalic dogs. However, MVE/Ei was higher in brachycephalic dogs after surgery than in non-brachycephalic dogs, suggesting diastolic dysfunction of the interventricular septum and higher left ventricular pressures.

One major limitation of our study is its small sample size, which included three different breeds of brachycephalic dogs at various stages of BOAS. In addition, the median follow-up time after surgical treatment was 9 months, and because cardiac remodeling is a chronic process, the lack of a further long-term follow-up is another limitation. The mean weight of the dogs before surgery was significantly lower than the mean weight of the dogs after surgery, and the mean weight of the non-brachycephalic group was significantly higher than that of the brachycephalic dogs before and after surgery. However, this difference in weight between the groups was limited by indexing weight-related variables.

Despite these limitations, BOAS patients in the study showed improvement of clinical symptoms, and right ventricular systolic and diastolic function appeared to improve. Similar to this study, studies in children have also found that cardiopulmonary changes caused by chronic adenotonsillar hypertrophy, such as hypoxemic pulmonary hypertension, *cor pulmonale*, and pulmonary edema, are reversible after adenotonsillectomy (18). Similarly, echocardiographic improvement has been noted in patients with OSA after therapy with continuous positive airway pressure (63). The study found significant echocardiographic differences between brachycephalic and non-brachycephalic dogs, with higher right heart pressures and lower systolic and diastolic ventricular function in brachycephalic dogs compared with non-brachycephalic dogs.

Future studies incorporating cardiac markers such as cardiac troponin I, C-reactive protein, and N-terminal pro B-type natriuretic peptide could further evaluate the changes that might occur in the myocardium with respect to damage, inflammation, or loading stress after surgical correction in dogs with BOAS. In addition, further follow-up of patients would help to fully evaluate the effects of rhinoplasty and folded-flap palatoplasty on cardiac morphology and function in the long term.

## Data availability statement

The raw data supporting the conclusions of this article will be made available by the authors, without undue reservation.

## Ethics statement

The animal study was reviewed and approved by Welfare Commission of the Veterinary Faculty, University of Ljubljana. Written informed consent was obtained from the owners for the participation of their animals in this study.

## Author contributions

ADP, ANS, and VE: study concepts and design. VE: surgery. ADP and MB: data acquisition and manuscript drafting. ANS: statistical analysis. ADP, ANS, MB, and VE: data analysis, interpretation, and manuscript revision for important intellectual content. All authors contributed to the article and approved the submitted version.

## Funding

The study was financially supported by the Slovenian Research Agency (grant no. P4-0053).

## Conflict of interest

The authors declare that the research was conducted in the absence of any commercial or financial relationships that could be construed as a potential conflict of interest.

## Publisher’s note

All claims expressed in this article are solely those of the authors and do not necessarily represent those of their affiliated organizations, or those of the publisher, the editors and the reviewers. Any product that may be evaluated in this article, or claim that may be made by its manufacturer, is not guaranteed or endorsed by the publisher.

## Abbreviations

Ai: Late diastolic annular velocity of the interventricular septum
Am: Late diastolic annular velocity of the left ventricular free wall
BOAS: Brachycephalic obstructive airway syndrome
CVCCI: Caudal vena cava collapsibility index
Ei: Early diastolic annular velocity of the interventricular septum
Em: Early diastolic annular velocity of the left ventricular free wall
GSLV4ch: Left ventricular global strain in apical long axis 4 chamber view
GSRV: Right ventricular global strain in apical long axis view
LALaxI: Diameter of left atrium in long axis index
LA/Ao: Ratio of the left atrium to aorta in short axis
MVE: Early diastolic mitral wave velocity
OSA: Obstructive sleep apnea
RVAsI: Right ventricular area in systole index
RVIDbasI: Right ventricular internal diameter just below the tricuspid annulus index
Si: Peak systolic annular velocity of the interventricular septum
